# Expanding the Role of Implantable Loop Recorders: Diagnostic and Therapeutic Yields Across Seven Clinical Indications in 388 Real-World Patients

**DOI:** 10.3390/jcm15051977

**Published:** 2026-03-05

**Authors:** Carlos Plappert, Philipp Lacour, Abdul S Parwani, Leif-Hendrik Boldt, Felix Bähr, Doreen Schöppenthau, Anna Feuerstein, Leonie H Wieland, Emanuel Heil, Felix Hohendanner, Nikolaos Dagres, Gerhard Hindricks, Ingo Hilgendorf, Florian Blaschke

**Affiliations:** 1 Department of Cardiology, Angiology and Intensive Care Medicine, German Heart Center at Charité, Campus Virchow Klinikum, 13353 Berlin, Germanyanna.feuerstein@dhzc-charite.de (A.F.);; 2 DZHK (German Centre for Cardiovascular Research), Partner Site Berlin, 10785 Berlin, Germany; 3 Kardiologie im Spreebogen, IB Hochschule Berlin, 12683 Berlin, Germany; 4 Charité-Universitätsmedizin Berlin, Corporate Member of Freie Universität Berlin and Humboldt-Universität zu Berlin, 10117 Berlin, Germany; 5 Department of Cardiology, Angiology and Intensive Care Medicine, German Heart Center at Charité, Campus Mitte, 10117 Berlin, Germany

**Keywords:** implantable loop recorder, diagnostic yield, syncope, presyncope, atrial fibrillation, atrial flutter, risk stratification

## Abstract

**Background/Objectives**: Implantable loop recorders (ILRs) enable long-term electrocadiographic monitoring and are established diagnostic tools for syncope and atrial fibrillation (AF). However, their diagnostic yield and therapeutic impact in other clinical settings remain less well defined. We aimed to evaluate the diagnostic yield and clinical impact of ILR implantation across contemporary clinical indications. **Methods**: In this retrospective single-center study, 388 patients who underwent ILR implantation between 2011 and 2018 were included. Indications were categorized into seven groups: unexplained syncope, presyncope, cryptogenic stroke or transient ischemic attack (TIA), AF detection, AF recurrence after atrial flutter (AFL) ablation, risk stratification in structural or inherited heart disease, and palpitations. **Results**: Among 388 patients (median age 63 [51.8–71.8] years, 57.5% male; median follow-up 17.0 [IQR 6.4–32.4] months), ILRs were most frequently implanted for syncope (44.6%), AF (20.4%), and stroke/TIA (12.9%). ILR-detected arrhythmias occurred in 241 patients (62.1%), with the highest detection rates in AF (83.5%) and AFL (73.7%). Indication-fulfilling diagnoses were established in 155 patients (39.9%), most frequently in AF (73.4%) and AFL (71.1%), after a median of 4.4 months (IQR 2.4–12.5). Nearly three quarters (72.9%) of diagnoses were made within the first year. ILR findings prompted therapeutic interventions in 156 patients (40.2%), including pacemaker implantation in syncope and rhythm- or anticoagulation-based therapies in AF. AF and AFL independently predicted higher diagnostic yield, while diagnostic yield and AF history predicted ILR-triggered therapy. AF, AFL, stroke/TIA, and AF history were associated with shorter time to first arrhythmia detection. Arrhythmia-free survival differed significantly across indication groups (*p* < 0.0001) and was lowest in AF and AFL, which demonstrated the highest cumulative incidence of indication-fulfilling arrhythmias. **Conclusions**: ILRs provide substantial diagnostic and therapeutic value across a broad range of indications. Beyond established uses in syncope and AF, clinically relevant yields were observed in presyncope, risk stratification, and AFL post-ablation, supporting broader consideration of ILRs and optimized patient selection.

## 1. Introduction

Implantable loop recorders (ILRs) allow prolonged, continuous single-lead electrocardigraphic (ECG) monitoring and have substantially shortened the time to diagnosis and treatment of many brady- and tachyarrhythmias. Over the past two decades, ILRs have become an essential tool for evaluating unexplained arrhythmia-related symptoms and detecting asymptomatic rhythm disorders [1,2].

In patients with recurrent syncope and inconclusive conventional work-up, the French healthcare system (FRESH) [3] and Early Use of an Implantable Loop Recorder in Syncope Evaluation: Assessment of Safety and Yield of the Approach to Syncope (EaSyAS II) [4] randomized trials have demonstrated the superiority of ILRs over conventional strategies. Overall, ILR implantation has been associated with a more than threefold higher likelihood of establishing a diagnosis compared with conventional evaluation [2]. Accordingly, early ILR implantation is strongly recommended in the 2018 European Society of Cardiology (ESC) Guidelines for syncope [5].

Beyond syncope, ILRs are increasingly used for continuous rhythm monitoring in patients with atrial fibrillation (AF), the most common sustained arrhythmia worldwide, associated with substantial morbidity and mortality [6,7]. ILRs facilitate early detection of previously unrecognized AF and recurrence after ablation, thereby guiding anticoagulation and antiarrhythmic therapy. The CRYptogenic STroke And underLying Atrial Fibrillation (CRYSTAL-AF) trial [8] demonstrated that long-term ILR monitoring detected AF in 30% of patients at three years compared to 3% with conventional follow-up, confirming its superiority and supporting current guideline recommendations [9].

Previous real-world studies have reported high diagnostic yields and low complication rates of ILRs across various clinical indications, with management changes observed in up to half of patients [10,11,12]. Nevertheless, their overall diagnostic and therapeutic impact has not been systematically evaluated in contemporary clinical practice. While ILRs are firmly established for certain indications such as unexplained syncope (ESC 2018, Class I, Level A [5]) and AF detection or post-stroke monitoring (American Heart Association [AHA]/American Stroke Association [ASA] 2021 [9]; ESC 2024 AF Guidelines, Class IIa [13]), their role in other contexts—including presyncope (Class IIb), palpitations with inconclusive noninvasive testing (Class IIb), risk stratification in structural or inherited heart disease (weak recommendations), and particularly AFL post-ablation follow-up, which is not specifically addressed in current guidelines—remains less well defined. Accordingly, we conducted a study at our tertiary academic center to systematically evaluate the diagnostic yield and clinical impact of ILR implantation across seven key clinical indications in contemporary practice.

## 2. Methods

### 2.1. Study Design

This retrospective, single-center study was conducted at the Department of Cardiology, Charité Campus Virchow Klinikum, a tertiary care university hospital in Berlin, Germany. The study evaluated the diagnostic yield and clinical impact of ILRs, focusing on the frequency and timing of diagnostic findings explaining the initial implantation indication and subsequent therapy initiation.

### 2.2. Study Participants

**Figure 1:**
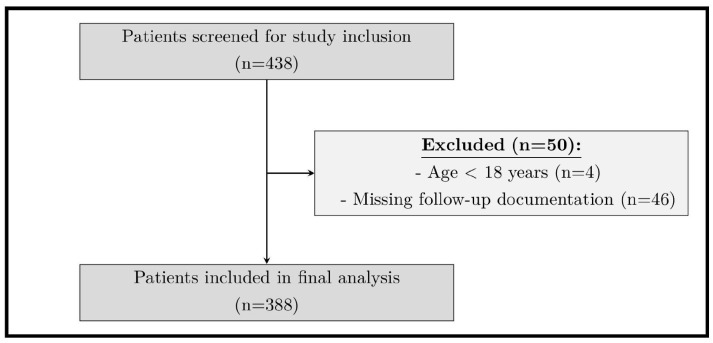
Patient flowchart showing screening, exclusions, and final study population.

A total of 438 patients aged ≥18 years who underwent ILR implantation between January 2011 and January 2018 were included. Exclusion criteria were age under 18 years at the time of ILR implantation and absence of follow-up within the cardiology department. Written informed consent was obtained from all patients. The study was approved by the institutional ethics committee and conducted in accordance with the Declaration of Helsinki. The patient selection process, including screening, exclusions, and final cohort size, is illustrated in Figure 1.

### 2.3. Patient Data Collection, Follow-Up, and Clinical Outcomes

#### 2.3.1. Data Collection and Baseline Characteristics

Data were collected for all eligible patients, including sociodemographic information, medical history, comorbidities, laboratory results, electrocardiogram (ECG) and echocardiographic findings, and current medications.

#### 2.3.2. ILR Implantation and Follow-Up Procedures

ILR implantation was performed in a left pectoral subcutaneous position under local anesthesia, with additional mild sedation using midazolam when required. Infection prophylaxis was administered according to institutional standards.

The following devices from three manufacturers were used: Medtronic (Minneapolis, MN, USA), Biotronik (Berlin, Germany), and St. Jude Medical (Saint Paul, MN, USA), including the models Reveal XT, Reveal DX, Reveal LINQ, BioMonitor, BioMonitor 2, SJM Confirm, and SJM Confirm RX. Patient follow-up was performed at the cardiology outpatient clinic of Charité Campus Virchow Klinikum by residents and board-certified cardiologists. ILRs were reviewed during scheduled visits or via remote monitoring (RM) when available, and patients were advised to present if symptoms occurred. All ILR-detected events were reviewed within a standardized institutional clinical workflow and adjudicated according to uniform clinical criteria before being classified as diagnostic endpoints. The median follow-up duration was 17.0 months (IQR 6.4–32.4 months). The observation period extended from ILR implantation to explantation or, at the latest, until 1 January 2018.

#### 2.3.3. Clinical Data Extraction and Study Endpoints

Clinical data were obtained from the institutional electronic medical record system (SAP, Charité, Berlin, Germany), including medical reports, echocardiographic findings, ILR implantation and explantation records, pacemaker and ICD implantation notes, electrophysiology reports, and ILR follow-up documentation.

The primary objective was to determine the diagnostic yield of ILRs, defined as the proportion of patients in whom a diagnosis explaining the initial implantation indication was established, along with the time to diagnosis and subsequent therapy initiation when applicable. Secondary analyses evaluated incidentally detected arrhythmias and their therapeutic implications.

### 2.4. Echocardiographic and ECG Assessment

Echocardiographic and ECG evaluations followed the established echocardiographic guidelines [14] and ESC recommendations [15]. LVEF was measured using the modified Simpson’s method, and ECGs closest to ILR implantation were analyzed for standard parameters.

### 2.5. Indications and Arrhythmia Classification

Indications for ILR implantation were classified into seven categories: (1) unexplained syncope, (2) unexplained presyncope, (3) cryptogenic stroke or transient ischemic attack (TIA), (4) detection of atrial fibrillation (AF), (5) AF detection after atrial flutter ablation, (6) arrhythmic risk stratification in patients with structural or inherited heart disease, and (7) palpitations or vegetative symptoms suggestive of arrhythmia. Presyncope was analyzed as a separate indication in accordance with current ESC syncope [5] classifications and differences in clinical presentation and symptom–rhythm correlation profiles compared with transient loss of consciousness. The risk stratification group comprised patients undergoing ILR implantation for long-term arrhythmia surveillance rather than symptom-driven diagnosis and included hypertrophic cardiomyopathy (HCM; n = 3), long QT syndrome (n = 1), Brugada syndrome (n = 2), unexplained wide-complex tachycardia (n = 3), and cardiac involvement in Fabry disease (n = 1) or transthyretin amyloidosis (ATTR; n = 1). In patients with HCM, mean interventricular septal thickness was modest, reflecting predominantly non-obstructive or mild phenotypes.

Arrhythmias were classified according to standard electrophysiological definitions, including atrioventricular block (second- or third-degree), atrioventricular nodal reentrant tachycardia (AVNRT/AVRT), AF, supraventricular tachycardia (SVT), sinus node dysfunction (sinus arrest, sinoatrial block, tachycardia–bradycardia syndrome), sinus bradycardia (<40 bpm), pauses >3 s, and ventricular tachycardia (VT), both sustained and non-sustained. For bradyarrhythmias (AV block and sinus node dysfunction), only events meeting contemporary guideline-based criteria for permanent PM implantation were considered clinically relevant diagnostic endpoints.

An indication was considered fulfilled when an ILR-detected arrhythmia was consistent with the original implantation indication, taking into account symptom–rhythm correlation as well as arrhythmia frequency and duration. In cases without arrhythmia detection but with therapy modification (e.g., discontinuation of anticoagulation), the indication was likewise considered fulfilled.

### 2.6. Therapeutic Management

Therapeutic management following ILR-detected arrhythmias included PM or ICD implantation, catheter ablation, left atrial appendage closure, or medical therapy such as initiation, adjustment, or discontinuation of antiarrhythmic drugs (AADs) or oral anti-coagulation (OAC), depending on arrhythmia type, patient characteristics, and symptom burden.

Therapeutic interventions were initiated based on ILR-detected arrhythmias in the clinical context and according to current guideline recommendations. OAC was initiated after AF detection in patients with an appropriate CHA_2_DS_2_-VASc risk profile. AADs or catheter ablation was considered for symptomatic atrial arrhythmias, while VT was regarded as clinically relevant if sustained or associated with symptoms or high-risk structural heart disease. In all cases requiring therapeutic intervention, ILR findings directly informed management decisions by confirming, newly detecting, or excluding clinically relevant arrhythmias.

### 2.7. Data Analysis and Statistical Methods

Continuous variables were tested for normality using the Shapiro-Wilk test and reported as mean *±* standard deviation (SD) or as median with interquartile range (IQR), as appropriate. Categorical data were presented as counts and percentages.

Group comparisons were performed using the Student’s *t*-test or one-way ANOVA for normally distributed continuous variables, and the Wilcoxon rank-sum test or Kruskal–Wallis test for non-normally distributed variables. Categorical variables were compared using the Chi-square (*χ*^2^) test or Fisher’s exact test, as appropriate. Therapeutic interventions were analyzed both per patient (unique count) and per indication category; therefore, totals across indication groups may exceed the number of treated patients.

Kaplan–Meier estimates with log-rank tests were used to analyze arrhythmia-free survival (time to first ILR-detected arrhythmia) across indication groups at 1-year and 2-year intervals. Arrhythmia probabilities were calculated for both time points, and patients without an arrhythmia at the last available follow-up were censored.

Univariate and multivariate logistic and Cox regression analyses were performed to identify predictors of diagnostic yield, ILR-triggered therapeutic interventions, and time to first diagnostic arrhythmia. Variables with *p* < 0.10 in univariate analysis or considered clinically relevant were included in the multivariate models.

For sensitivity analyses addressing potential heterogeneity in device generation and monitoring workflow, additional multivariable logistic regression models were adjusted for indication group, remote monitoring (RM) status, manufacturer, and follow-up duration. To ensure model stability and avoid sparse-data bias, individual device models were consolidated into three manufacturer groups (Medtronic, Biotronik, and Abbott/St. Jude). The number of events per variable was assessed to minimize the risk of model overfitting.

Results are presented as odds ratios (OR) or hazard ratios (HR) with 95% confidence intervals (CI) and corresponding *p*-values. All analyses were performed using R statistical software (version 4.5.2). A two-tailed *p*-value < 0.05 was considered statistically significant. No adjustment for multiple comparisons was performed; therefore, *p*-values should be interpreted as exploratory.

## 3. Results

### 3.1. Baseline Characteristics

Of 438 screened patients, 50 (11.4%) were excluded due to age under 18 years (n = 4) and missing follow-up documentation (n = 46). Thus, 388 patients were included in the final analysis (median [IQR] age 63 [51.8–71.8] years; 223 [57.5%] male). Baseline characteristics are shown in Table 1. The median BMI was 26.0 (IQR 23.6–29.1) kg/m^2^. Arterial hypertension (60.6%), dyslipidemia (35.1%), and CAD (22.0%) were the most frequent comorbidities. AF was present in 114 patients (29.4%), while AFL and VT were observed in 15.2% and 5.7%, respectively. A total of 152 patients (39.2%) were followed via a remote monitoring (RM) system (Biotronik Home Monitoring, Medtronic CareLink, St. Jude Medical Merlin.net).

**Figure 2:**
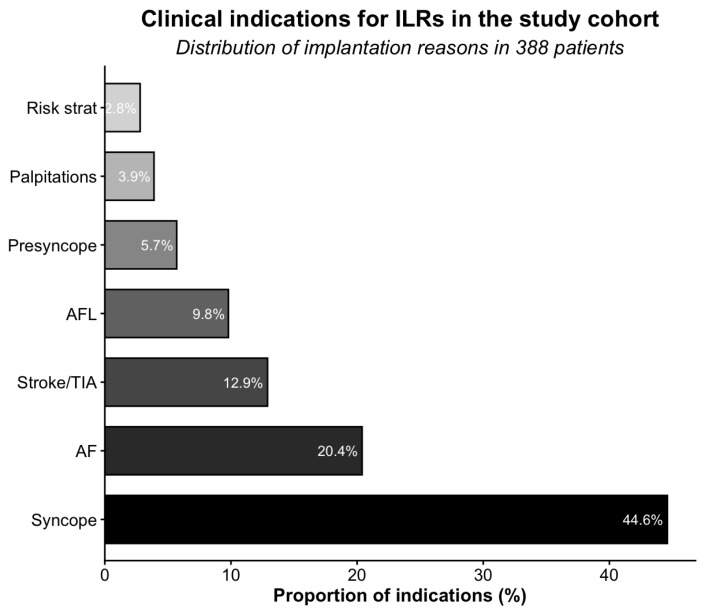
Clinical indications for ILRs among 388 patients. Abbreviations: AF, atrial fibrillation; AFL, atrial flutter; Risk strat, risk stratification; TIA, transient ischemic attack.

The median LVEF was 55% (IQR 55–60%). Syncope was the leading indication for ILR implantation (44.6%), followed by AF (20.4%) and cryptogenic stroke/TIA (12.9%) (Figure 2).

Overall, the cohort reflects a real-world ILR population with a high burden of cardiovascular risk factors and preserved LVEF. Details on implantation characteristics, complications, and device management are provided in the Supplementary Material (Figure S1).

### 3.2. Diagnostic Value of ILR

#### 3.2.1. Kaplan–Meier Analysis of Arrhythmia-Free Survival by ILR Indication

**Figure 3:**
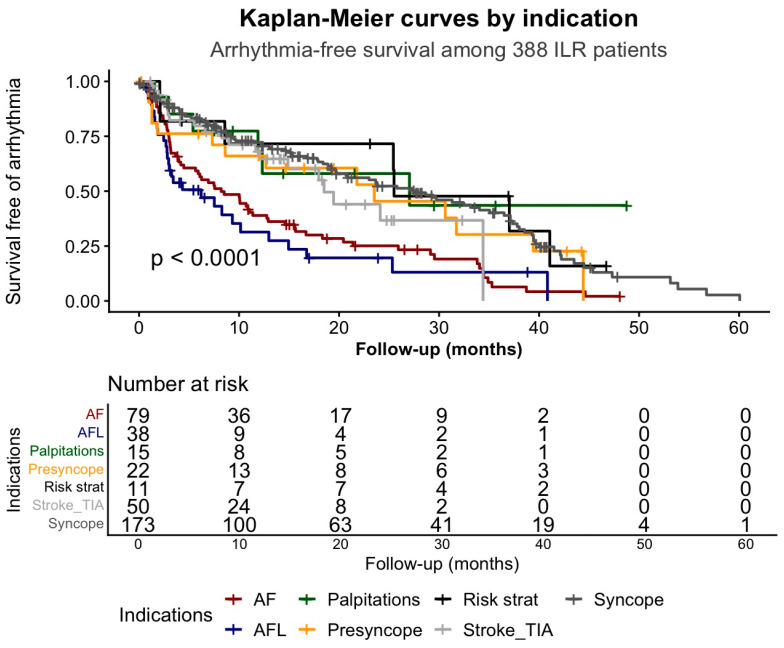
Kaplan–Meier estimates of arrhythmia-free survival stratified by ILR indication.

Arrhythmia-free survival differed significantly across the seven ILR indication groups (log rank test, *p* < 0.001) (Figure 3).

After 12 months, arrhythmia-free survival was 38.9% (95% CI 29.3–51.7) in the AF group and 31.4% (95% CI 18.4–53.5) in the AFL group. In contrast, higher rates were observed in patients with palpitations [67.7% (95% CI 45.7–100)], presyncope [66.0% (95% CI 48.4–90.1)], risk stratification [71.6% (95% CI 48.8–100)], stroke/TIA [68.1% (95% CI 54.9–84.5)], and syncope [72.2% (95% CI 65.4–79.7)].

At 24 months, arrhythmia-free survival further declined to 25.1% (95% CI 16.7–37.7) and 19.6% (95% CI 9.2–41.8) in the AF and AFL groups, respectively. Patients with palpitations, presyncope, and stroke/TIA showed intermediate rates [58.0% (95% CI 35.4–95.2); 45.4% (95% CI 26.6–77.5); 44.1% (95% CI 28.1–69.1)], whereas those with risk stratification [71.6% (95% CI 48.8–100)] and syncope [52.3% (95% CI 44.3–61.8)] maintained the highest arrhythmia-free survival.

Consequently, AF- and AFL-related ILR implantation showed the highest early arrhythmia detection, while patients undergoing ILR for risk stratification or syncope largely remained arrhythmia-free throughout follow-up (Table 2). These results highlight distinct temporal patterns of event occurrence across indication groups.

The event was defined as the first ILR-detected arrhythmia of any type. Time zero was the date of ILR implantation. Patients were censored at the time of last follow-up, ILR explantation, or 1 January 2018, whichever occurred first. This analysis reflects the time to first arrhythmia detection irrespective of the primary diagnostic endpoint. AF, atrial fibrillation; AFL, atrial flutter; Risk strat, risk stratification; TIA, transient ischemic attack.

#### 3.2.2. Kaplan–Meier Estimates of Cumulative Incidence of Indication-Fulfilling Diagnoses by ILR Indication

**Figure 4:**
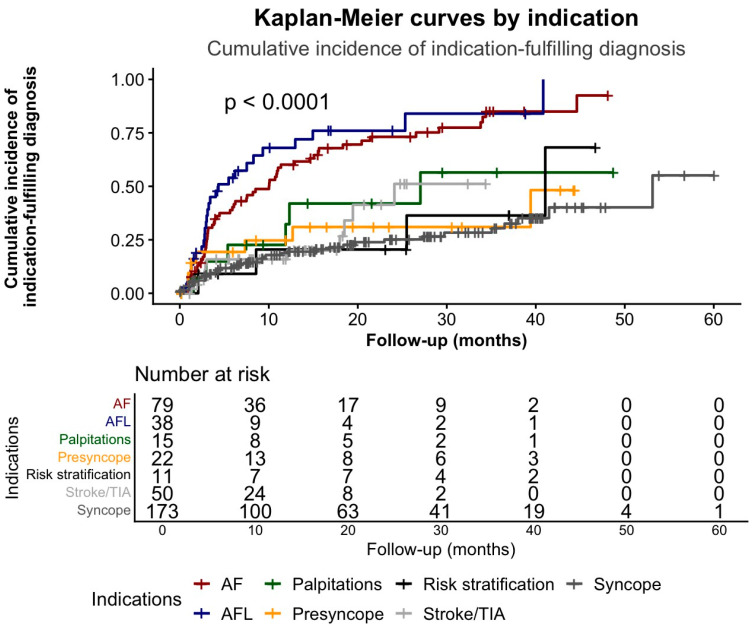
Kaplan–Meier estimates of cumulative incidence (1–S[t]) of indication-fulfilling diagnoses stratified by ILR indication.

While arrhythmia-free survival differed significantly across indication groups (Figure 3), the cumulative incidence of indication-fulfilling diagnoses showed a similarly pronounced separation (Figure 4).

The event was defined as the first arrhythmia fulfilling the initial implantation indication, including diagnostic clarification when applicable. Time zero was the date of ILR implantation. Patients were censored at the time of last follow-up, ILR explantation, or 1 January 2018, whichever occurred first. AF, atrial fibrillation; AFL, atrial flutter; TIA, transient ischemic attack.

#### 3.2.3. ILR-Detected Arrhythmias Stratified by Clinical Indication

An ILR-detected arrhythmia occurred in 241 of 388 patients (62.1%). The median time to first arrhythmia detection was 3.4 months (IQR 2.0–10.2). Detection tended to occur earlier in patients with presyncope, stroke/TIA, and AFL indications and later in those implanted for risk stratification or palpitations; however, these differences were not statistically significant (*p* = 0.39) (Table 3).

The distribution of ILR-detected arrhythmias differed significantly across indication groups (*p* < 0.001) (Table 4). AF was the most frequent arrhythmia overall and was most commonly detected in patients implanted for AF (49 of 79 patients, 62.0%) and in those implanted after AFL ablation (22 of 38 patients, 57.9%; *p* < 0.001). In patients with syncope, AF (16.8%), AV block (7.0%), and SSS (4.6%) were the most frequent findings. In contrast, presyncope patients most commonly showed ES (22.7%), AF (18.2%), and VT (9.1%). In patients implanted for palpitations, ES was the most frequent arrhythmia (20.0%), followed by AV block, AVNRT, SVT, and sinus bradycardia (each 6.7%). In stroke/TIA patients, AF (12.0%) and SVT (8.0%) predominated.

AV block (n = 12, 7.0%; *p* = 0.40) and SSS (n = 8, 4.6%; *p* = 0.75) were numerically more frequent in syncope patients but did not differ significantly across groups. VT was uncommon but occurred more frequently in risk stratification and presyncope groups (*p* < 0.05). ES followed a similar pattern (*p* < 0.05). Other arrhythmias (pauses/asystole, AVNRT, SVT, and sinus bradycardia) were infrequent and did not differ significantly across indication groups (*p* > 0.20).

#### 3.2.4. Arrhythmias Fulfilling the Diagnostic Indication

**Figure 5:**
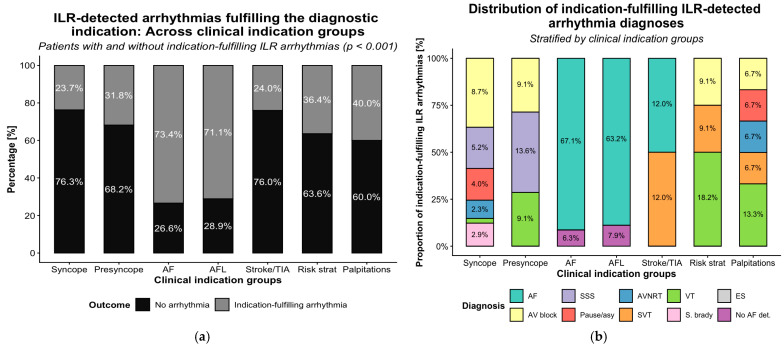
Indication-fulfilling arrhythmias of ILR monitoring across clinical indication groups. (**a**) Proportion of patients with ILR-detected arrhythmias; (**b**) distribution of indication-fulfilling diagnoses. Only proportions > 0.5% are shown. AF, atrial fibrillation; AFL, atrial flutter; AV, atrioventricular; AVNRT, atrioventricular nodal reentrant tachycardia; ES, extrasystole; No AF det., no atrial fibrillation detected; Pause/asy, pause or asystole; Risk strat, risk stratification; S. brady, sinus bradycardia; SSS, sick sinus syndrome; SVT, supraventricular tachycardia; TIA, transient ischemic attack; VT, ventricular tachycardia.

An arrhythmia fulfilling the diagnostic indication for ILR implantation was detected in 147 patients (37.9%). In an additional eight patients (2.1%), the absence of arrhythmia—specifically, no AF despite clinical suspicion—provided diagnostic clarification and guided further management. Thus, ILR monitoring fulfilled or clarified the initial diagnostic indication in 155 patients (39.9%) (Figure 5a). The median time to indication-fulfilling arrhythmia was 4.4 months (IQR 2.4–12.5). Of the 155 indication-fulfilling diagnoses, 72.9% were detected within the first 12 months after ILR implantation (range across indications 50.0–85.2%). Early diagnostic yield was highest in atrial arrhythmia-related indications (AF: 75.9%, AFL: 85.2%) and lowest in stroke/TIA (58.3%) and risk stratification (50.0%). The diagnostic yield differed significantly across indication groups (*p* < 0.001) (Figure 5b, Table 5). AF was the most frequent arrhythmia fulfilling the diagnostic indication in patients implanted for AF (n = 53, 67.1%) and in those implanted after AFL ablation (n = 24, 63.2%; *p* < 0.001). In contrast, bradyarrhythmias predominated among patients with syncope, where AV block (n = 15, 8.7%) and SSS (n = 9, 5.2%) represented the main indication-fulfilling diagnoses (*p* < 0.01 and *p* < 0.05, respectively). SVT occurred more often in stroke/TIA patients (n = 6 [12.0%]; *p* < 0.001), whereas VT was rare but more frequent in risk stratification and palpitations groups (*p* < 0.001). Bradyarrhythmias, including sinus bradycardia and pauses, were infrequent and showed no significant group differences (*p* > 0.20). In selected AF and AFL patients, absence of AF detection (6.3% and 7.9%) was itself diagnostically relevant (*p* < 0.01).

#### 3.2.5. ILR-Triggered Therapeutic Interventions

Overall, 156 patients (40.2%) underwent at least one therapeutic intervention following ILR detection. A total of 172 procedures were performed, indicating that some patients underwent multiple treatments; 16 additional interventions (10.3%) occurred in patients receiving more than one therapy.

Across the entire cohort, AAD therapy was the most frequent intervention (n = 59), followed by PM implantation (n = 47), PVI (n = 32), and initiation of OAC (n = 30), reflecting patient-based totals across all indication groups.

Of the treated patients, 123 (31.7%) had indication-fulfilling arrhythmias, whereas 33 (8.5%) received therapy for clinically relevant non-indication-fulfilling findings (Table 5). Within the subset of indication-fulfilling ILR findings, PM implantation was the most frequent procedure (n = 40), occurring predominantly in patients with syncope (*p* < 0.001). AAD therapy (n = 35) and PVI (n = 25) were mainly performed in AF patients and differed significantly across indication groups (*p* < 0.01 and *p* < 0.001, respectively). Initiation of OAC (n = 28) was observed primarily in AF and AFL patients (*p* < 0.001). Other interventions, including CTI ablation and ECV, were less frequent but also varied significantly across indication groups.

#### 3.2.6. Symptoms During ILR-Detected Events

Clinical symptoms during ILR-detected arrhythmias varied significantly across indication groups (Table 6). Most symptomatic events were recorded in patients with syncope (n = 35) or AF (n = 15). Syncope during an ILR-detected event was reported in 28 patients with a syncope indication (16.2%), representing the highest rate across all groups (*p* < 0.001). Presyncope occurred in three patients from the presyncope group (13.6%; *p* < 0.01). Vegetative symptoms (e.g., dizziness or nausea) were most frequent in patients with palpitations (33.3%, *p* < 0.001). Palpitations as a concomitant symptom were predominantly reported in AFL (13.2%) and AF (10.2%) indications (*p* < 0.001). Asymptomatic events were common, particularly in AFL (42.1%) and AF (29.1%) patients (*p* < 0.001).

### 3.3. Integrated Diagnostic and Therapeutic Outcomes by Indication

To provide a consolidated overview of diagnostic yield and ILR-triggered therapeutic consequences across clinical indications, Table 7 summarizes patient-based outcomes across indication groups.

### 3.4. Predictors of ILR-Outcomes

#### 3.4.1. Predictors of Diagnostic Yield

**Figure 6:**
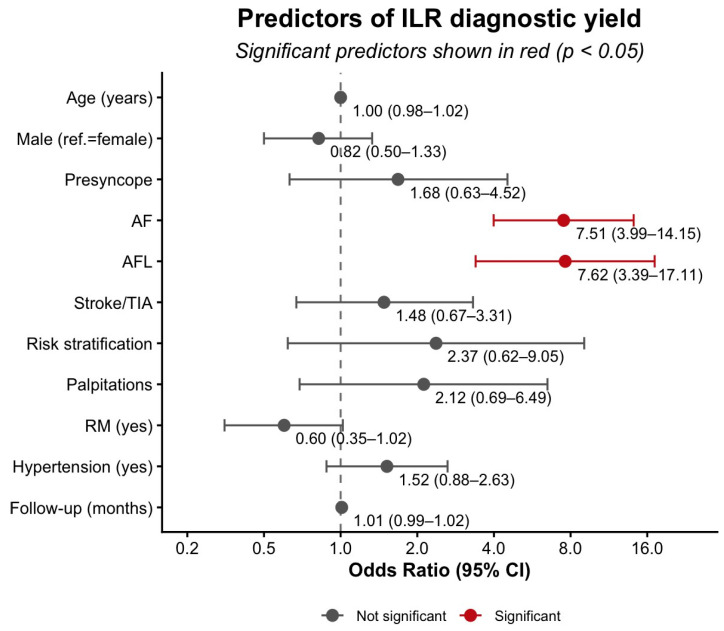
Multivariate analysis of ILR diagnostic yield. Abbreviations: AF, atrial fibrillation; AFL, atrial flutter; ref., reference category (female as reference for sex); RM, remote monitoring; TIA, transient ischemic attack.

In univariate analyses, AF and AFL indications were associated with higher ILR diagnostic yield (AF: OR = 8.89, 95% CI 4.83–16.36; *p* < 0.001; AFL: OR = 7.90, 95% CI 3.61–17.30; *p* < 0.001). RM was associated with lower ILR diagnostic yield (OR = 0.42, 95% CI 0.27–0.64; *p* < 0.001), possibly related to shorter median follow-up (11.8 [4.9–19.8] vs. 17.0 [6.4–32.4] months) and a higher proportion of AF indications. Arterial hypertension was also associated with higher ILR diagnostic yield (OR = 2.01, 95% CI 1.31–3.09; *p* < 0.01). In the multivariate model, only AF (adjusted OR = 7.51, 95% CI 3.99–14.15; *p* < 0.001) and AFL (adjusted OR = 7.62, 95% CI 3.39–17.11; *p* < 0.001) remained independently associated with higher ILR diagnostic yield. The association of RM was attenuated and no longer statistically significant after adjustment (adjusted OR = 0.60, 95% CI 0.35–1.02; *p* = 0.06). Age, sex, hypertension, and follow-up duration were not independently associated with ILR diagnostic yield (all *p* > 0.10). Figure 6 and Supplementary Table S1 summarize these findings.

#### 3.4.2. Predictors of ILR-Triggered Therapeutic Interventions

In univariate analyses, AF indication (OR = 2.57, 95% CI 1.49–4.44; *p* < 0.001), diagnostic yield (OR = 12.64, 95% CI 7.76–20.62; *p* < 0.001), and AF history (OR = 4.85, 95% CI 3.04–7.75; *p* < 0.001) were associated with a higher likelihood of ILR-triggered therapeutic interventions, whereas tachyarrhythmia indication (OR = 0.32, 95% CI 0.15–0.68; *p* < 0.01) and RM (OR = 0.33, 95% CI 0.21–0.52; *p* < 0.001) were associated with a lower likelihood.

**Figure 7:**
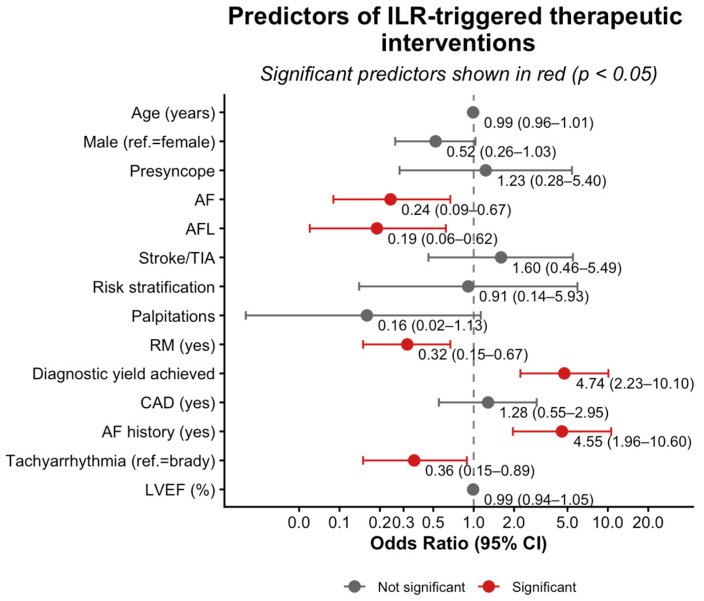
Multivariate analysis of ILR-triggered therapeutic interventions. Abbreviations: AF, atrial fibrillation; AFL, atrial flutter; CAD, coronary artery disease; LVEF, left ventricular ejection fraction; ref., reference category (female as reference for sex); RM, remote monitoring; TIA, transient ischemic attack.

In the multivariate model, the occurrence of an ILR-detected diagnostic arrhythmia (adjusted OR = 4.74, 95% CI 2.23–10.10; *p* < 0.001) and a history of AF (adjusted OR = 4.55, 95% CI 1.96–10.60; *p* < 0.001) remained independent predictors of therapeutic interventions. Conversely, AF indication (adjusted OR = 0.24, 95% CI 0.09–0.67; *p* < 0.01), AFL indication (adjusted OR = 0.19, 95% CI 0.06–0.62; *p* < 0.01), RM (adjusted OR = 0.32, 95% CI 0.15–0.67; *p* < 0.01), and tachyarrhythmia indication (adjusted OR = 0.36, 95% CI 0.15–0.89; *p* < 0.05) were associated with a lower likelihood of interventions. Age, sex, and LVEF were not significant predictors (all *p* > 0.10). Figure 7 and Supplementary Table S2 summarize these findings.

#### 3.4.3. Predictors of Time to First Diagnostic Arrhythmia

In univariate analyses, AF indication (HR = 2.30, 95% CI 1.67–3.15; *p* < 0.001), AFL indication (HR = 2.88, 95% CI 1.88–4.42; *p* < 0.001), hypertension (HR = 1.71, 95% CI 1.30–2.26; *p* < 0.001), AF history (HR = 1.71, 95% CI 1.32–2.22; *p* < 0.001), older age (HR = 1.01, 95% CI 1.00–1.02; *p* < 0.05), and lower LVEF (HR = 0.97, 95% CI 0.95–0.99; *p* < 0.05) were associated with a shorter time to the first diagnostic arrhythmia. Other factors, including sex, presyncope, stroke/TIA, risk stratification, palpitations indication, RM, DM, and CAD, showed no significant association (all *p* > 0.05).

**Figure 8:**
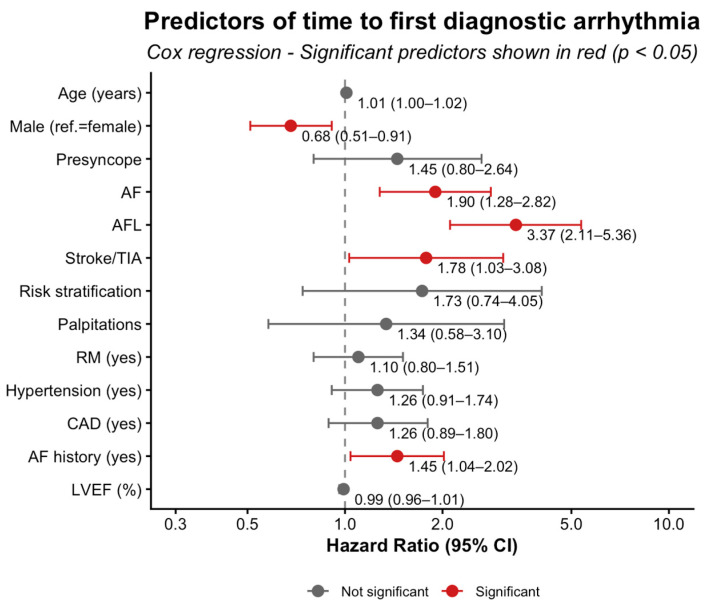
Cox regression analysis of time to first diagnostic arrhythmia. Abbreviations: AF, atrial fibrillation; AFL, atrial flutter; CAD, coronary artery disease; LVEF, left ventricular ejection fraction; ref., reference category (female as reference for sex); RM, remote monitoring; TIA, transient ischemic attack.

In the multivariate Cox model, AFL indication (adjusted HR = 3.37, 95% CI 2.11–5.36; *p* < 0.001), AF indication (adjusted HR = 1.90, 95% CI 1.28–2.82; *p* < 0.01), stroke/TIA indication (adjusted HR = 1.78, 95% CI 1.03–3.08; *p* < 0.05), and AF history (adjusted HR = 1.45, 95% CI 1.04–2.02; *p* < 0.05) remained independent predictors of shorter time to first diagnostic arrhythmia. Conversely, male sex (adjusted HR = 0.68, 95% CI 0.51–0.91; *p* < 0.01) was independently associated with a longer time to event. Age, RM, hypertension, CAD, and LVEF were not significant predictors in the adjusted model (all *p* > 0.10). Figure 8 and Supplementary Table S3 summarize these findings.

Sensitivity analyses were performed to evaluate the robustness of findings with respect to RM workflow and device manufacturer (Supplementary Tables S4–S8). Indication group remained the primary determinant of diagnostic yield and ILR-triggered therapy after adjustment for manufacturer group, RM status, and follow-up duration. RM was independently associated with lower odds of ILR-detected events and therapeutic interventions, whereas follow-up duration was associated with the probability of event detection but did not materially alter therapy outcomes. Findings were consistent across manufacturer groups, supporting robustness to device heterogeneity.

## 4. Discussion

This study offers a comprehensive real-world assessment of ILR performance across seven clinical indications in 388 patients, demonstrating clear differences in diagnostic yield, event timing, and clinical management. The main findings are as follows:

1. ILRs provided substantial diagnostic (39.9%) and therapeutic value (40.2%) across a wide range of clinical indications.
2. Diagnostic yield was highest in AF (73.4%) and AFL (71.1%), with arrhythmias detected after a median of 3–4 months; both arrhythmias were the strongest independent predictors in all regression models.
3. In syncope, ILRs primarily identified bradyarrhythmias, frequently leading to targeted interventions such as PM implantation.

### 4.1. Implantation Indications

The distribution of implantation indications in our cohort—syncope (44.6%), AF detection (20.4%), and stroke/TIA (12.9%)—is broadly consistent with prior studies. In the Hellenic ILR Registry, involving 871 patients, syncope/presyncope was the leading indication (61.9%), followed by palpitations (10.4%) and AF detection after cryptogenic stroke (27.7%) [10]. Similarly, in a cohort of 312 patients, Padmanabhan et al. found syncope (66%), palpitations (16%), and cryptogenic stroke (8.7%) as the dominant indications [11]. In a multicenter cohort of 386 patients, Ibrahim et al. observed a predominance of syncope (84.8%), with palpitations (12.8%) and suspected AF (11.7%) [12].

These studies confirm a consistent pattern across healthcare systems: syncope remains the primary driver of ILR implantation, with AF detection and stroke/TIA serving as key secondary indications. Although palpitations were included in several cohorts, they were typically analyzed less systematically and not as a primary indication. In contrast, our study evaluates seven clinically relevant ILR use cases—including presyncope, risk stratification, AFL post-ablation follow-up, and palpitations—providing a more comprehensive picture of contemporary ILR utilization [10,11,12]. This broader scope enables a more nuanced understanding of diagnostic yield and event timing across heterogeneous patient groups and highlights several understudied clinical scenarios in which ILRs appear increasingly valuable.

### 4.2. Diagnostic Yield

ILRs consistently increase the diagnostic yield of rhythm disorders [10]. In our cohort, 39.9% of patients received a diagnosis that met or clarified the initial indication.

The diagnostic yield for **syncope or presyncope** typically ranges between 30% and 50% [10,16]. The Randomized Assessment of Syncope Trial (RAST) [17] showed higher diagnostic rates with ILRs than with conventional evaluation (55% vs. 19%), while the PICTURE study [16] reported that ILRs clarified the mechanism of syncope in 30% of patients within one year. The EaSyAS II trial [4] demonstrated that remote ILR monitoring shortens time to ECG diagnosis and reduces additional testing. In our cohort, 23.7% of patients received an indication-fulfilling diagnosis, most commonly AV block (8.7%) and SSS (5.2%). This distribution closely mirrors the findings of Letsas et al., who likewise identified SSS (18.6%) and AV block (5.0%) as the predominant diagnoses [10].

ILRs also provide substantial diagnostic value in **suspected AF detection**. In CRYSTAL-AF [8], the ILR-guided AF detection rate reached 12.4% at 12 months compared with 2% in the conventional arm. In our cohort, 73.4% of patients received an ILR-based diagnosis, predominantly AF (67.1%) or rule-out of AF (6.3%). This exceeds the 40% diagnostic yield for AF detection reported by Ibrahim et al. [12].

Prolonged **rhythm monitoring after stroke** significantly increases AF detection rates [18]. Similarly, in the PER DIEM trial [19], AF was identified in 15.3% of ILR-monitored patients compared with 4.7% using an external loop recorder. A meta-analysis of eight studies confirmed both higher AF detection and increased anticoagulation use [20]. Importantly, ILR monitoring was associated with reduced recurrent stroke risk [21]. In our cohort, 24.0% of patients had indication-fulfilling diagnoses—most commonly AF (12.0%) and SVT (12.0%)—closely aligning with real-world findings (AF 19.5%, SVT 1.7%) [10]. Evidence on ILR diagnostic yield for **palpitations** is more limited, though available data support its superiority over conventional testing [10,22]. In the Recurrent Unexplained Palpitations (RUP) study [23], diagnostic yield reached 73% with ILRs vs. 21% with standard evaluation. In our cohort, the diagnostic yield was 40%. Unlike Letsas et al., who reported a predominance of SVTs, VT (13%) was the most frequent diagnosis.

Evidence for **presyncope** as a stand-alone indication is limited [10], and data on ILR diagnostic yield in **risk stratification [24]** and **AFL post-ablation [25]** remain sparse. Our study adds important insights, showing a high diagnostic yield in AFL follow-up (71.1%) and moderate yields in risk stratification (36.4%) and presyncope (31.8%). Prior ILR studies after cavotricuspid isthmus ablation similarly reported high AF incidences of 55–85% [25,26,27,28], consistent with our AFL cohort. Across all regression models, AF and AFL consistently emerged as the strongest independent predictors of diagnostic yield and time to event, reinforcing their role as the most impactful ILR indications in contemporary practice. Our findings align with Pistelli et al., who observed higher diagnostic yields in recurrent presyncope compared with isolated syncope [29]. Likewise, Sakhi et al. reported heterogeneous yields ranging from 14% in inherited primary arrhythmia syndrome (IPAS) to 60% in structural heart disease (SHD) [24], and our 36.4% yield falls within this spectrum, reflecting the mixed composition of our cohort (IPAS and SHD).

Kaplan–Meier estimates showed that 1-year diagnostic rates were highest in AFL and AF. Diagnostic rates for syncope (27.8%), stroke/TIA (31.9%), and palpitations (32.3%) exceeded those reported by Letsas et al. (22.5%, 12.4%, and 13.3%, respectively) [10]. These differences likely reflect a higher pre-test probability for arrhythmias in our cohort and a more indication-specific definition of diagnostic yield. Together, these findings show that ILR performance varies substantially across indications and underscore the importance of appropriate patient selection.

### 4.3. Therapeutic Implications

ILR monitoring led to therapeutic interventions in 40.2% of patients, confirming its substantial clinical impact. This aligns with previously reported management changes of 47% [11]. Overall, 156 patients underwent ILR-guided therapy, including 172 procedures, indicating that 10.3% required multiple interventions. Of these, 31.7% were treated for arrhythmias fulfilling the initial indication, while 8.5% received therapy for previously unsuspected but clinically relevant rhythm disturbances. Thus, ILRs frequently detect actionable events that directly alter patient management [11].

**Syncope** showed a high therapeutic intervention rate, with ILR-guided therapy performed in 37.6% of patients. PM or ICD implantation was required in 19.1% of patients due to clinically significant bradyarrhythmias. This closely aligns with prior real-world evidence, with Padmanabhan et al. reporting 19% and Letsas et al. 26.1% device implantation in syncope patients [10,11].

**AF detection** showed the strongest therapeutic impact in our cohort, with ILR-guided therapy performed in 60.8%. ILR-guided indication-fulfilling therapies most frequently included PVI (27.8%), AAD and OAC initiation (26.6% both). These findings mirror randomized and real-world ILR studies, in which newly detected AF frequently prompted anticoagulation and, in selected patients, rhythm-control strategies such as antiarrhythmic medication and catheter ablation [8,19,21,25].

**AFL** showed a high therapeutic intervention rate, with ILR-guided therapy performed in 36.8% of patients, mainly initiation of AAD and OAC (10.5% each) and ECV (7.9%). Similarly, Attanasio et al. reported frequent rhythm-control interventions after ILR-detected AF following CTI ablation, including PVI in 14% and ECV in 6% [25], supporting the strong clinical utility of ILRs in atrial tachyarrhythmia management.

In our cohort, repeat CTI ablation was not part of the predefined AFL follow-up strategy. This likely reflects the post-ablation nature of this indication, as most patients had undergone prior successful CTI ablation and ILR implantation was primarily intended for detection of subsequent AF. During follow-up, only four patients were diagnosed with recurrent atrial flutter. When typical AFL was documented, repeat cavotricuspid isthmus ablation was performed according to clinical indication. Overall, ILR monitoring predominantly identified AF rather than recurrent typical AFL, thereby favoring AF-directed therapies such as PVI, AAD initiation, or anticoagulation.

**Stroke/TIA monitoring** led to ILR-guided therapy in 26.0% of patients, with nearly all indication-fulfilling diagnoses resulting in treatment. Interventions included AAD and OAC initiation (4.0% both). Diagnostic events were evenly split between AF and SVT (each 12.0%), indicating that actionable arrhythmias in secondary stroke prevention extend beyond AF alone. This aligns with Letsas et al., who reported AF detection in 19.5% with anticoagulation in all cases [10], while our data additionally emphasize the therapeutic importance of SVT in this setting.

**Palpitations** resulted in ILR-guided therapy in 20.0% of patients, including ECV, EPS, and AAD initiation (each 6.7%), reflecting a broad actionable spectrum. Although three patients (20.0%) in the palpitations group had ILR-detected ES, these findings were not classified as indication-fulfilling arrhythmias. Only arrhythmias deemed clinically relevant and consistent with the implantation indication were included in the indication-fulfilling analysis. These results align with real-world data from Padmanabhan et al., who reported ILR-triggered interventions in approximately 25% of patients with unexplained palpitations [11].

**Presyncope** showed substantial therapeutic benefit, with at least one ILR-guided intervention in 40.9% of patients—higher than the 26.1% previously reported [10].

**Risk stratification** demonstrated notable impact, with at least one ILR-guided intervention in 36.4% of patients (PM/ICD implantation, or AAD initiation, each 9.1%). Similar ILR-triggered therapies were reported previously, including ICD implantation in 10% of patients with SHD [24]. In patients with a history of AVNRT or VT, ILR implantation was selected in cases without a clear indication for immediate invasive electrophysiological evaluation. These patients presented with infrequent symptoms, uncertain arrhythmia burden, or non-sustained events, making prolonged rhythm monitoring a reasonable diagnostic strategy. ILRs allowed continuous surveillance and symptom–rhythm correlation while avoiding potentially unnecessary invasive procedures.

These results provide quantitative support for ILR utility in underrepresented indications. Taken together, these findings underscore the substantial clinical utility of ILRs as decision-guiding tools across diverse indications. Even in groups with modest diagnostic yield, the therapeutic relevance per detected event was high, highlighting the prognostic significance and management implications of ILR-guided diagnosis.

### 4.4. Implications for Guideline Recommendations

Beyond the guideline-supported indications for syncope, AF detection, and post-stroke monitoring [5,9,13], our findings identify additional scenarios in which ILRs provide substantial diagnostic value. **Presyncope**, despite its current Class IIb recommendation [5], achieved a higher diagnostic yield than syncope, challenging the assumption of lower arrhythmic risk. **AFL post-ablation**, not addressed in current guidelines, demonstrated high diagnostic utility and was a strong independent predictor of event detection. **Risk stratification** likewise showed diagnostic performance. These findings suggest that several underrepresented indications merit stronger future guideline recommendations, given the early arrhythmia detection and actionable diagnostic clarity ILRs provide.

### 4.5. Study Limitations and Strengths

This retrospective single-center design introduces potential selection and treatment biases and limits generalizability. Pre-implant diagnostic evaluation was not standardized and relied on physician judgment, which may have influenced indication assignment and pre-test probability. Variability in ILR programming, RM workflows, and provider responses to device alerts could have affected arrhythmia detection and therapy decisions. Although follow-up was adequate, longer monitoring might have identified additional events, particularly low-burden AF. Symptom–rhythm correlation was limited by incomplete documentation, restricting assessment of the symptomatic relevance of detected arrhythmias.

Implantation-related complications occurred in 7 patients (1.8%), including device malposition with pain (n = 3), pocket infection (n = 3), and post-procedural hematoma (n = 1). This rate is lower than previously reported (3.3%) [12] and supports the overall safety profile of ILR implantation in routine practice. Although ILR implantations were performed between 2011 and 2018, follow-up was shorter because monitoring ended after diagnostic clarification, device explantation, battery depletion, loss to follow-up, or at the predefined end of the observation period in January 2018. Technological advances during the study period may have influenced diagnostic yield, particularly through device miniaturization and RM capabilities. However, all devices provided continuous rhythm surveillance consistent with routine clinical practice, and overall diagnostic performance reflects real-world ILR use across successive device generations.

Nevertheless, this study provides robust indication-specific performance data from a large unselected real-world cohort, supporting more refined ILR patient selection.

### 4.6. Clinical Implications and Conclusion

ILRs provide meaningful diagnostic and therapeutic value across a broad range of indications in routine practice. Beyond established uses in syncope and AF detection, we observed clinically relevant yields in presyncope and risk stratification, and a particularly high yield in AFL post-ablation. Importantly, most clinically relevant diagnoses were established early during follow-up, especially in AF and AFL indications, supporting early ILR implantation while highlighting the importance of optimized patient selection to maximize clinical impact.

## Figures and Tables

**Table 1:** Baseline characteristics of the study population.

Characteristics	Overall Study Population (n = 388)	Missing
Age, median (IQR), y	62.8 (51.8–71.7)	
Sex, n (%)		
Male	223 (57.5)	
Female	165 (42.5)	
Body mass index, kg/m^2^	26.0 (23.6–29.1)	2
**Echocardiographic characteristics**		
Left ventricular ejection fraction, %	55.0 (55.0–60.0)	16
Right ventricular ejection fraction, %	55.0 (55.0–55.0)	49
Left ventricular septal diameter, mm	12 (10–13)	98
**Laboratory, median (IQR)**		
Estimated GFR	82.0 (67.0–90.0)	18
Hemoglobin, g/dL	13.7 (12.9–14.6)	8
**Medical history, n (%)** *Rhythm disorders*		
Atrial fibrillation	114 (29.4)	
Atrial flutter	59 (15.2)	
Atrial tachycardia	14 (3.6)	
Ventricular tachycardia	22 (5.7)	
Paroxysmal supraventricular tachycardia	2 (0.5)	
AVNRT	18 (4.6)	
Bradycardic rhythm disorders	11 (2.8)	
*Others*		
Coronary artery disease	85 (22.0)	
Obesity (BMI > 30 kg/m^2^)	88 (22.7)	2
Hypertension	235 (60.6)	
Dyslipidemia	136 (35.1)	
Stroke	82 (21.1)	
Diabetes mellitus	57 (14.7)	
**Electrocardiogram characteristics**		
Atrioventricular block, n (%)	36 (9.3)	
Left bundle branch block, n (%)	15 (3.9)	
Right bundle branch block, n (%)	33 (8.5)	
**Medications, n (%)**		
Statins	179 (46.1)	
Direct oral anticoagulants	78 (20.1)	
Vitamin-K antagonists	78 (20.1)	
Acetylsalicylic acid	149 (38.4)	
ACE inhibitor	139 (35.8)	
Angiotensin II receptor blocker	88 (22.7)	
Aldosterone antagonists	19 (4.9)	
Diuretics	111 (28.6)	
Antiarrhythmic drugs	11 (2.8)	
Beta-blocker	213 (54.9)	
Calcium channel blocker	77 (19.8)	

Continuous variables are presented as median (interquartile range) and categorical variables as n (%). AVNRT, atrioventricular nodal reentrant tachycardia; eGFR, estimated glomerular filtration rate (CKD-EPI, mL/min/1.73 m^2^); n, number.

**Table 2:** Arrhythmia-free survival rates at 1 and 2 years by ILR indication.

Groups	1-Year	95% CI	2-Years	95% CI
Atrial fibrillation	38.9%	29.3–51.7	25.1%	16.7–37.7
Atrial flutter	31.4%	18.4–53.5	19.6%	9.2–41.8
Palpitations	67.7%	45.7–100	58.0%	35.4–95.2
Presyncope	66.0%	48.4–90.1	45.4%	26.6–77.5
Risk stratification	71.6%	48.8–100	71.6%	48.8–100
Stroke/TIA	68.1%	54.9–84.5	44.1%	28.1–69.1
Syncope	72.2%	65.4–79.7	52.3%	44.3–61.8

Data are presented as Kaplan–Meier estimates with 95% confidence intervals. TIA, transient ischemic attack.

**Table 3:** Time to first ILR-detected arrhythmia by clinical indication.

Clinical Indication	Median (Months)	IQR	n (Patients with Events)
Syncope	3.99	1.80–10.0	100
Stroke/TIA	2.96	2.35–13.6	20
Risk stratification	8.57	2.09–19.5	7
Presyncope	2.83	1.42–3.16	14
Palpitations	8.64	3.56–12.2	6
Atrial flutter	2.99	1.63–6.54	28
Atrial fibrillation	3.78	2.62–10.4	66

Data are presented as median (interquartile range). n, number; TIA, transient ischemic attack.

**Table 4:** ILR-detected arrhythmias stratified by clinical indication during follow-up.

Characteristic (Patients)	Syncope (n = 173, 44.6%)	Presyncope (n = 22, 5.7%)	AF (n = 79, 20.4%)	AFL (n = 38, 9.8%)	Stroke/TIA (n = 50, 12.9%)	Risk Strat (n = 11, 2.8%)	Palpitations (n = 15, 3.9%)	*p*-Value
≥1 ILR event, n (%)	100 (57.8)	14 (63.6)	66 (83.5)	28 (73.7)	20 (40.0)	7 (63.6)	6 (40.0)	<0.001
Diagnosis								
AF	29 (16.8)	4 (18.2)	49 (62.0)	22 (57.9)	6 (12.0)	0	0	<0.001
AV block	12 (7.0)	1 (4.5)	2 (2.5)	0	3 (6.0)	1 (9.1)	1 (6.7)	0.40
SSS	8 (4.6)	1 (4.5)	1 (1.3)	1 (2.6)	3 (6.0)	0	0	0.75
Pause/asystole	7 (4.0)	0	1 (1.3)	0	0	0	0	0.67
AVNRT	4 (2.3)	1 (4.5)	0	0	2 (4.0)	0	1 (6.7)	0.24
SVT	13 (7.5)	0	5 (6.3)	1 (2.6)	4 (8.0)	1 (9.1)	1 (6.7)	0.78
VT	5 (2.9)	2 (9.1)	0	1 (2.6)	0	2 (18.2)	0	<0.05
Sinus bradycardia	7 (4.0)	0	0	0	1 (2.0)	0	1 (6.7)	0.31
ES	15 (8.7)	5 (22.7)	8 (10.1)	3 (7.9)	1 (2.0)	3 (27.3)	3 (20.0)	<0.05
No AF detected	0	0	0	0	0	0	0	n/a

Data are presented as n (% of patients within each indication group). Percentages refer to patients and may exceed 100% across categories due to multiple arrhythmias per patient. Abbreviations: AF, atrial fibrillation; AFL, atrial flutter; AV, atrioventricular; AVNRT, atrioventricular nodal reentrant tachycardia; ES, extrasystole; SSS, sick sinus syndrome; SVT, supraventricular tachycardia; TIA, transient ischemic attack; VT, ventricular tachycardia; n/a, not applicable.

**Table 5:** Indication-fulfilling ILR-detected arrhythmias and related therapeutic interventions stratified by clinical indication.

Characteristic	Syncope (n = 173, 44.6%)	Presyncope (n = 22, 5.7%)	AF (n = 79, 20.4%)	AFL (n = 38, 9.8%)	Stroke/TIA (n = 50, 12.9%)	Risk Strat (n = 11, 2.8%)	Palpitations (n = 15, 3.9%)	*p*-Value
Indication-fulfilling events, n (%)	41 (23.7)	7 (31.8)	58 (73.4)	27 (71.1)	12 (24.0)	4 (36.4)	6 (40.0)	<0.001
Diagnosis								
AF	0	0	53 (67.1)	24 (63.2)	6 (12.0)	0	0	<0.001
AV block	15 (8.7)	2 (9.1)	0	0	0	1 (9.1)	1 (6.7)	<0.01
SSS	9 (5.2)	3 (13.6)	0	0	0	0	0	<0.05
Pause/asystole	7 (4.0)	0	0	0	0	0	1 (6.7)	0.21
AVNRT	4 (2.3)	0	0	0	0	0	1 (6.7)	0.35
SVT	0	0	0	0	6 (12.0)	1 (9.1)	1 (6.7)	<0.001
VT	1 (0.6)	2 (9.1)	0	0	0	2 (18.2)	2 (13.3)	<0.001
Sinus bradycardia	5 (2.9)	0	0	0	0	0	0	0.63
ES	0	0	0	0	0	0	0	n/a
No AF detected	0	0	5 (6.3)	3 (7.9)	0	0	0	<0.01
Intervention types								
PM implantation	32 (18.5)	4 (18.2)	3 (3.8)	0	0	1 (9.1)	0	<0.001
ICD implantation	1 (0.6)	1 (4.5)	0	0	0	1 (9.1)	0	0.07
PVI	1 (0.6)	0	22 (27.8)	2 (5.3)	0	0	0	<0.001
CTI	0	0	5 (6.3)	0	1 (2.0)	0	0	<0.05
ECV	0	0	3 (3.8)	3 (7.9)	0	0	1 (6.7)	<0.05
LAA occlusion	0	0	1 (1.3)	0	0	0	0	0.55
Atrial ablation (other)	3 (1.7)	0	6 (7.6)	1 (2.6)	0	0	0	0.46
EPS	1 (0.6)	1 (4.5)	0	0	1 (2.0)	0	1 (6.7)	0.19
Antiarrhythmic drugs	4 (2.3)	2 (9.1)	21 (26.6)	4 (10.5)	2 (4.0)	1 (9.1)	1 (6.7)	<0.01
OAC	1 (0.6)	0	21 (26.6)	4 (10.5)	2 (4.0)	0	0	<0.001

Data are presented as n (% of patients within each indication group). Therapeutic interventions are reported on a per-patient basis. Abbreviations (alphabetical): AF, atrial fibrillation; AFL, atrial flutter; AV, atrioventricular; AVNRT, atrioventricular nodal reentrant tachycardia; CTI, cavotricuspid isthmus ablation; ECV, electrical cardioversion; EPS, electrophysiological study; ICD, implantable cardioverter-defibrillator; LAA, left atrial appendage; OAC, oral anticoagulation; PM, pacemaker; SSS, sick sinus syndrome; SVT, supraventricular tachycardia; TIA, transient ischemic attack; VT, ventricular tachycardia.

**Table 6:** Symptoms at the time of ILR-detected arrhythmia stratified by clinical indication.

Symptoms (During Arrhythmia)	Syncope (n = 173, 44.6%)	Presyncope (n = 22, 5.7%)	AF (n = 79, 20.4%)	AFL (n = 38, 9.8%)	Stroke/TIA (n = 50, 12.9%)	Risk Strat (n = 11, 2.8%)	Palpitations (n = 15, 3.9%)	*p*-Value
Presyncope	1 (0.6)	3 (13.6)	0	0	0	0	0	<0.01
Syncope	28 (16.2)	1 (4.5)	2 (2.5)	0	0	1 (9.1)	0	<0.001
Vegetative	5 (2.9)	2 (9.1)	5 (6.3)	1 (2.6)	0	1 (9.1)	5 (33.3)	<0.001
Palpitations	1 (0.6)	0	8 (10.2)	5 (13.2)	3 (6.0)	0	1 (6.7)	<0.001
Asymptomatic	17 (9.8)	1 (4.5)	23 (29.1)	16 (42.1)	7 (14.0)	2 (18.2)	0	<0.001
Not documented or unknown	121 (69.9)	15 (68.2)	41 (51.9)	16 (42.1)	40 (80.0)	7 (63.6)	9 (60.0)	<0.01

Data are presented as n (% of patients within each indication group).

**Table 7:** Decision-impact summary of ILR monitoring stratified by clinical indication.

Indication	N	FU (Median, Months)	≥1 ILR Event (%)	Fulfilled (%)	Time to 1st Event (mo)	Time to Fulfilling Diagnosis (mo)	≥1 Therapy (%)	Device (%)	Rhythm (%)	OAC (%)
Syncope	173	15.3 (5.9–31.3)	57.8	23.7	3.99	6.31 (2.14–16.8)	37.6	19.1	18.5	3.5
Presyncope	22	15.6 (3.0–31.5)	63.6	31.8	2.83	1.84 (1.12–10.0)	40.9	22.7	22.7	0
AF	79	30.9 (16.1–41.3)	83.5	73.4	3.78	5.78 (2.82–11.3)	60.8	6.3	44.3	26.6
AFL	38	12.6 (6.3–20.2)	73.7	71.1	2.99	3.12 (2.14–6.87)	36.8	2.6	26.3	10.5
Stroke/TIA	50	11.8 (5.1–18.4)	40.0	24.0	2.96	2.96 (2.64–18.1)	26.0	8.0	14.0	4.0
Risk stratification	11	25.4 (15.5–37.0)	63.6	36.4	8.57	17.0 (6.95–29.4)	36.4	18.2	18.2	0
Palpitations	15	21.6 (6.5–32.6)	40.0	40.0	8.64	8.64 (3.56–12.2)	20.0	0	13.3	0

Data include diagnostic yield, time to first ILR-detected arrhythmia, time to indication-fulfilling diagnosis, and ILR-triggered therapeutic interventions.

## Data Availability

All data generated or analyzed during this study are included in this published article. Further inquiries can be directed to the corresponding author.

## Supplementary Materials

The following supporting information can be downloaded at: https://www.mdpi.com/article/10.3390/jcm15051977/s1, Figure S1: Distribution of ILR devices among 388 patients. Values indicate the number and percentage of implantations for each device model; Table S1: Univariate and multivariate logistic regression analyses for predictors of ILR diagnostic yield; Table S2: Univariate and multivariate logistic regression analyses for predictors of ILR-triggered therapeutic interventions; Table S3: Univariate and multivariate Cox regression analyses for predictors of time to first diagnostic arrhythmia. Table S4: Indication-specific outcomes stratified by remote monitoring (RM); Table S5: Manufacturer grouping applied for multivariable regression analyses to ensure model stability and avoid sparse-data bias; Table S6: Multivariable logistic regression model for indication-fulfilling diagnosis. Reference groups: AF (indication), Medtronic (manufacturer), No RM; Table S7: Multivariable logistic regression model for ≥1 ILR-detected event. Reference groups: AF (indication), Medtronic (manufacturer), No RM. Adjusted for indication group, manufacturer group, remote monitoring (RM), and follow-up duration; Table S8: Multivariable logistic regression model for ≥1 ILR-triggered therapy. Reference groups: AF (indication), Medtronic (manufacturer), No RM.
