# Supplementary material for: Expanding the Role of Implantable Loop Recorders: Diagnostic and Therapeutic Yields Across Seven Clinical Indications in 388 Real-World Patients
Source: J Clin Med. 2026 Mar 5;15(5):1977. doi: 10.3390/jcm15051977 (PMC12986014; doi:10.3390/jcm15051977)
Supplement: Supplementary file 1 [file jcm-15-01977-s001.zip › jcm-4131868-SI.pdf]

Supplementary Material

Expanding the Role of Implantable Loop Recorders: Diagnostic and Therapeutic Yields Across Seven Clinical Indications in 388 Real-World Patients

Carlos Plappert, Philipp Lacour, Abdul S Parwani, Leif-Hendrik Boldt, Felix Bähr, Doreen Schöppenthau, Anna Feuerstein, Leonie H Wieland, Emanuel Heil, Felix Hohendanner, Nikolaos Dagres, Gerhard Hindricks, Ingo Hilgendorf, Florian Blaschke

SUPPLEMENTARY TEXT

Implantation and management of ILRs

Implantation characteristics

A total of 388 ILRs were implanted. Medtronic devices represented the majority (Reveal LINQ, n = 124 [32.0%]; Reveal XT, n = 144 [37.1%]; Reveal DX, n = 28 [7.2%]), followed by St. Jude Medical Confirm systems (SJM Confirm, n = 47 [12.1%]; Confirm RX, n = 3 [0.8%]) and Biotronik Biomonitor devices (Biomonitor, n = 22 [5.7%]; Biomonitor 2-AF, n = 20 [5.2%]).

Implantation-related complications

Implantation-related complications occurred in 7 patients (1.8%), including device malposition with pain (n = 3, 42.9%), pocket infection (n = 3, 42.9%), and post-procedural hematoma (n = 1, 14.3%).

Device explantation

ILRs were explanted in 133 patients (34.3%). The median time to explantation was 26.0 ± 16.8 months (IQR 8.2-60.3). The most frequent reasons were PM implantation (n = 53, 13.7%) and battery depletion (n = 47, 12.1%), followed by patient request (n = 24, 6.2%), revision due to complications (n = 4, 1.0%), and absence of arrhythmia detection during follow-up (n = 2, 0.5%). In two cases (0.5%), the reason for explantation was not specified.

Device replacement

ILRs were replaced in 22 patients (5.7%). The median time to replacement was 33.3 ± 17.5 months (IQR 27.8-44.4). The most frequent reasons were battery depletion (n = 16, 72.7%) and revision due to complications (n = 3, 13.6%), followed by device malfunction such as missing recordings or memory exhaustion due to artifacts (n = 2, 9.1%), and patient request (n = 1, 4.5%).

SUPPLEMENTARY FIGURE

Supplementary Figure S1: **Distribution of ILR devices among 388 patients. Value indicate the number and percentage of implantations for each device model.**

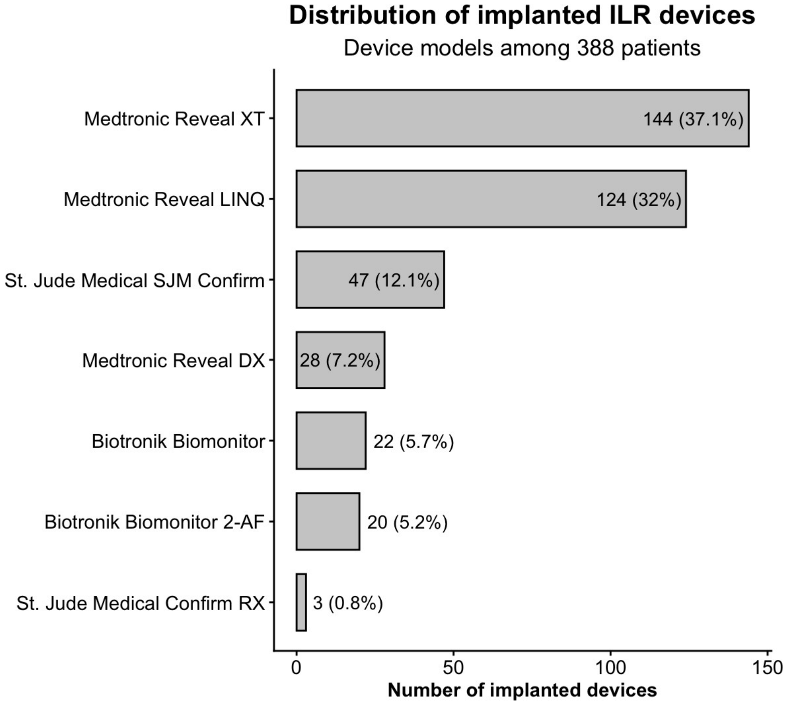

SUPPLEMENTARY TABLES

Supplementary Table S1: **Univariate and multivariate logistic regression analyses for predictors of ILR diagnostic yield.**

Predictor	Univariate OR (95% CI)	p-value	Multivariate OR (95% CI)	p-value
Age (years)	1.01 (1.00-1.03)	0.08	1.00 (0.98-1.02)	1.00
Sex (female = ref.)	1.00 (ref.)			
Male	1.04 (0.69-1.57)	0.85	0.82 (0.50-1.33)	0.41
Syncope (ref.)	1.00 (ref.)			
Presyncope indication	1.50 (0.57-3.94)	0.41	1.68 (0.63-4.52)	0.30
AF indication	8.89 (4.83-16.36)	<0.001	7.51 (3.99-14.15)	**<0.001**
AFL indication	7.90 (3.61-17.30)	<0.001	7.62 (3.39-17.11)	**<0.001**
Stroke/TIA indication	1.02 (0.49-2.13)	0.96	1.48 (0.67-3.31)	0.33
Risk stratification	1.84 (0.51-6.60)	0.35	2.37 (0.62-9.05)	0.21
Palpitations indication	2.15 (0.72-6.39)	0.17	2.12 (0.69-6.49)	0.19
RM (no = ref.)	1.00 (ref.)			
RM (yes)	0.42 (0.27-0.64)	<0.001	0.60 (0.35-1.02)	0.06
Hypertension (no = ref.)	1.00 (ref.)			
Hypertension (yes)	2.01 (1.31-3.09)	<0.01	1.52 (0.88-2.63)	0.14
CAD (no = ref.)	1.00 (ref.)		--	--
CAD (yes)	1.07 (0.65-1.74)	0.79	--	--
DM (no = ref.)	1.00 (ref.)		--	--
DM (yes)	1.11 (0.63-1.96)	0.72	--	--
Dyslipidemia (no = ref.)	1.00 (ref.)		--	--
Dyslipidemia (yes)	1.37 (0.90-2.09)	0.15	--	--
LVEF (%)	0.99 (0.96-1.03)	0.67	--	--
Follow-up (months)	1.02 (1.01-1.03)	<0.01	1.01 (0.99-1.02)	0.37

Abbreviations: AF, atrial fibrillation; AFL, atrial flutter; CAD, coronary artery disease; CI, confidence interval; DM, diabetes mellitus; LVEF, left ventricular ejection fraction; OR, odds ratio; ref, reference; RM, remote monitoring; TIA, transient ischemic attack.

Supplementary Table S2: **Univariate and multivariate logistic regression analyses for predictors of ILR-triggered therapeutic interventions.**

**Predictor**	**Univariate OR (95% CI)**	**p-value**	**Multivariate OR (95% CI)**	**p-value**
Age (years)	1.01 (1.00-1.02)	0.15	0.99 (0.96-1.01)	0.30
Sex (female = ref.)	1.00 (ref.)		1.00 (ref.)	
Male	0.82 (0.54-1.23)	0.33	0.52 (0.26-1.03)	0.06
Syncope (ref.)	1.00 (ref.)		1.00 (ref.)	
Presyncope indication	1.15 (0.47-2.84)	0.76	1.23 (0.28-5.40)	0.78
AF indication	2.57 (1.49-4.44)	<0.001	0.24 (0.09-0.67)	**<0.01**
AFL indication	0.97 (0.47-2.01)	0.93	0.19 (0.06-0.62)	**<0.01**
Stroke/TIA indication	0.58 (0.29-1.18)	0.13	1.60 (0.46-5.49)	0.46
Risk stratification	0.95 (0.27-3.37)	0.94	0.91 (0.14-5.93)	0.92
Palpitations indication	0.42 (0.11-1.53)	0.19	0.16 (0.02-1.13)	0.07
RM (no = ref.)	1.00 (ref.)		1.00 (ref.)	
RM (yes)	0.33 (0.21-0.52)	<0.001	0.32 (0.15-0.67)	<0.01
Diagnostic yield achieved (no = ref.)	1.00 (ref.)		1.00 (ref.)	
Diagnostic yield (yes)	12.64 (7.76-20.62)	<0.001	4.74 (2.23-10.10)	**<0.001**
CAD (no = ref.)	1.00 (ref.)		1.00 (ref.)	
CAD (yes)	1.19 (0.73-1.94)	0.48	1.28 (0.55-2.95)	0.57
AF history (no = ref.)	1.00 (ref.)		1.00 (ref.)	
AF history (yes)	4.85 (3.04-7.75)	<0.001	4.55 (1.96-10.60)	**<0.001**
Arrhythmia category (bradycardia = ref.)	1.00 (ref.)		1.00 (ref.)	
Tachycardia (yes)	0.32 (0.15-0.68)	<0.01	0.36 (0.15-0.89)	<0.05
LVEF (%)	1.00 (0.97-1.04)	0.93	0.99 (0.94-1.05)	0.81

Abbreviations: AF, atrial fibrillation; AFL, atrial flutter; CAD, coronary artery disease; CI, confidence interval; LVEF, left ventricular ejection fraction; OR, odds ratio; ref, reference; RM, remote monitoring; TIA, transient ischemic attack.

Supplementary Table S3: **Univariate and multivariate Cox regression analyses for predictors of time to first diagnostic arrhythmia.**

**Predictor**	**Univariate HR (95% CI)**	**p-value**	**Multivariate HR (95% CI)**	**p-value**
Age (years)	1.01 (1.00-1.02)	<0.05	1.01 (1.00-1.02)	0.24
Sex (female = ref.)	1.00 (ref.)		1.00 (ref.)	
Male	0.96 (0.74-1.24)	0.75	0.68 (0.51-0.91)	<0.01
Syncope (ref.)	1.00 (ref.)		1.00 (ref.)	
Presyncope indication	1.18 (0.67-2.07)	0.56	1.45 (0.80-2.64)	0.22
AF indication	2.30 (1.67-3.15)	<0.001	1.90 (1.28-2.82)	**<0.01**
AFL indication	2.88 (1.88-4.42)	<0.001	3.37 (2.11-5.36)	**<0.001**
Stroke/TIA indication	1.25 (0.76-2.04)	0.38	1.78 (1.03-3.08)	**<0.05**
Risk stratification	0.90 (0.42-1.93)	0.78	1.73 (0.74-4.05)	0.21
Palpitations indication	0.84 (0.37-1.92)	0.68	1.34 (0.58-3.10)	0.50
RM (no = ref.)	1.00 (ref.)		1.00 (ref.)	
RM (yes)	0.96 (0.72-1.27)	0.76	1.10 (0.80-1.51)	0.55
Hypertension (no = ref.)	1.00 (ref.)		1.00 (ref.)	
Hypertension (yes)	1.71 (1.30-2.26)	<0.001	1.26 (0.91-1.74)	0.16
DM (no = ref.)	1.00 (ref.)		--	--
DM (yes)	1.13 (0.78-1.63)	0.51	--	--
CAD (no = ref.)	1.00 (ref.)		1.00 (ref.)	
CAD (yes)	1.24 (0.92-1.66)	0.16	1.26 (0.89-1.80)	0.20
AF history (no = ref.)	1.00 (ref.)		1.00 (ref.)	
AF history (yes)	1.71 (1.32-2.22)	<0.001	1.45 (1.04-2.02)	**<0.05**
LVEF (%)	0.97 (0.95-0.99)	<0.05	0.99 (0.96-1.01)	0.32

Abbreviations: AF, atrial fibrillation; AFL, atrial flutter; CAD, coronary artery disease; CI, confidence interval; DM, diabetes mellitus; HR, hazard ratio; LVEF, left ventricular ejection fraction; ref, reference; RM, remote monitoring; TIA, transient ischemic attack.

Supplementary Table S4: **Indication-specific outcomes stratified by remote monitoring (RM).**

Indication	RM	N	% Any Event	% Fulfilled	% Any Therapy
AF	No RM	62	85.5	79.0	67.7
AF	RM	17	76.5	52.9	35.3
AFL	No RM	31	83.9	74.2	38.7
AFL	RM	7	28.6	57.1	28.6
Palpitations	No RM	8	50.0	50.0	37.5
Palpitations	RM	7	28.6	28.6	0.0
Presyncope	No RM	13	69.2	38.5	46.2
Presyncope	RM	9	55.6	22.2	33.3
Risk stratification	No RM	5	80.0	40.0	60.0
Risk stratification	RM	6	50.0	33.3	16.7
Stroke/TIA	No RM	8	50.0	25.0	37.5
Stroke/TIA	RM	42	38.1	23.8	23.8
Syncope	No RM	109	63.3	25.7	45.0
Syncope	RM	64	48.4	20.3	25.0

Abbreviations: AF, atrial fibrillation; AFL, atrial flutter; RM, remote monitoring; TIA, transient ischemic attack.

Supplementary Table S5: **Manufacturer grouping applied for multivariable regression analyses to ensure model stability and avoid sparse-data bias.**

Original Device Models	Manufacturer Group
Medtronic Reveal DX, Reveal XT, Reveal LINQ	Medtronic
Biotronik Biomonitor 2-AF	Biotronik
St. Jude Medical Confirm, Confirm RX	Abbott/St. Jude

Supplementary Table S6: **Multivariable logistic regression model for indication-fulfilling diagnosis. Reference groups: AF (indication), Medtronic (manufacturer), No RM.**

Variable	OR	95% CI	p-value
AFL	1.27	0.46–3.64	0.65
Palpitations	0.24	0.07–0.79	**0.020**
Presyncope	0.17	0.06–0.47	**0.001**
Risk stratification	0.20	0.05–0.77	**0.023**
Stroke/TIA	0.17	0.07–0.41	**<0.001**
Syncope	0.12	0.06–0.22	**<0.001**
RM	0.49	0.28–0.85	**0.012**
Abbott/St. Jude	0.66	0.26–1.57	0.35
Biotronik	2.52	1.22–5.23	**0.013**
FU (per month)	1.00	0.99–1.02	0.66

Supplementary Table S7: **Multivariable logistic regression model for ≥1 ILR-detected event. Reference groups: AF (indication), Medtronic (manufacturer), No RM. Adjusted for indication group, manufacturer group, remote monitoring (RM), and follow-up duration.**

Variable	OR	95% CI	p-value
AFL	0.43	0.14–1.30	0.13
Palpitations	0.18	0.05–0.59	**0.005**
Presyncope	0.47	0.16–1.42	0.17
Risk stratification	0.45	0.11–1.98	0.26
Stroke/TIA	0.26	0.10–0.61	**0.003**
Syncope	0.36	0.17–0.70	**0.003**
RM	0.54	0.33–0.90	**0.017**

Abbott/St. Jude	2.09	0.82–5.87	0.14
Biotronik	1.24	0.61–2.59	0.55
FU (per month)	1.02	1.00–1.03	**0.039**

Supplementary Table S8: **Multivariable logistic regression model for ≥1 ILR-triggered therapy. Reference groups: AF (indication), Medtronic (manufacturer), No RM.**

Variable	OR	95% CI	p-value
AFL	0.61	0.24–1.58	0.31
Palpitations	0.18	0.04–0.66	**0.016**
Presyncope	0.53	0.19–1.44	0.22
Risk stratification	0.45	0.11–1.71	0.25
Stroke/TIA	0.44	0.18–1.03	0.063
Syncope	0.44	0.25–0.79	**0.007**
RM	0.36	0.21–0.60	**<0.001**
Abbott/St. Jude	0.49	0.21–1.09	0.083
Biotronik	1.10	0.53–2.25	0.79
FU (per month)	1.01	1.00–1.02	0.21
